# Functional Identification of *Corynespora cassiicola*-Responsive miRNAs and Their Targets in Cucumber

**DOI:** 10.3389/fpls.2019.00668

**Published:** 2019-05-31

**Authors:** Xiangyu Wang, Guangchao Yu, Junyue Zhao, Na Cui, Yang Yu, Haiyan Fan

**Affiliations:** ^1^College of Bioscience and Biotechnology, Shenyang Agricultural University, Shenyang, China; ^2^College of Horticulture, Shenyang Agricultural University, Shenyang, China; ^3^Key Laboratory of Protected Horticulture of Ministry of Education, Shenyang Agricultural University, Shenyang, China

**Keywords:** cucumber, *Corynespora cassiicola*, microRNA, transient transformation, short tandem target mimic, lignin

## Abstract

Target leaf spot (TLS), which is caused by *Corynespora cassiicola* (*C. cassiicola*), is one of the most important diseases in cucumber (*Cucumis sativus* L.). Our previous research identified several *C. cassiicola*-responsive miRNAs in cucumber by high-throughput sequencing, including two known miRNAs and two novel miRNAs. The target genes of these miRNAs were related to secondary metabolism. In this study, we verified the interaction between these miRNAs and target genes by histochemical staining and fluorescence quantitative assays of GUS. We transiently expressed the candidate miRNAs and target genes in cucumber cotyledons to investigate the resistance to *C. cassiicola*. Transient expression of miR164d, miR396b, Novel-miR1, and Novel-miR7 in cucumber resulted in decreased resistance to *C. cassiicola*, while transient expression of *NAC* (inhibited by miR164d), *APE* (inhibited by miR396b), *4CL* (inhibited by Novel-miR1), and *PAL* (inhibited by Novel-miR7) led to enhanced resistance to *C. cassiicola*. In addition, overexpression of *4CL* and *PAL* downregulated lignin synthesis, and overexpression of Novel-miR1 and Novel-miR7 also downregulated lignin synthesis, indicating that the regulation of *4CL* and *PAL* by Novel-miR1 and Novel-miR7 could affect lignin content. The tobacco rattle virus (TRV) induced short tandem target mimic (STTM)-miRNA silencing vector was successfully constructed, and target miRNAs were successfully silenced. The identification of disease resistance and lignin content showed that silencing candidate miRNAs could improve cucumber resistance to *C. cassiicola*.

## Introduction

Cucumber (*Cucumis sativus* L.) target leaf spot (TLS) is caused by *Corynespora cassiicola* (*C. cassiicola*), which is an obligate oomycete pathogen (Teramoto et al., 2011; Li et al., 2012). TLS affects a wide range of cucumbers worldwide, including those in China (Li et al., 2012; Yang et al., 2012), the United States (Ishii et al., 2007), Japan (Miyamoto et al., 2010), and South Korea (Kwon et al., 2003). TLS is a foliar disease that can occur throughout the growing period of cucumber, but it is more serious in the middle and late stages of growth than in other stages (Wen et al., 2015). There are abundant variations in the size and structure of the *C. cassiicola* conidia, as well as the color of the colonies. These variations are reflected not only in different colonies of the same strain but also among strains of different geographical origins and different hosts (Nghia et al., 2008; Qi et al., 2011). Therefore, it is important to study the molecular mechanisms of cucumber resistance to *C. cassiicola* and find new resistance gene resources.

Plant microRNAs (miRNAs) are a class of small non-coding single stranded RNAs encoded by endogenous genes that are mainly involved in gene expression and regulation at the post-transcriptional level (Waterhouse and Hellens, 2015). In plants, miRNAs can function by interacting with target genes, mainly through degradation and inhibition of target mRNAs (Slack et al., 2000; Khraiwesh et al., 2012). Plants are often subjected to various environmental stresses during their growth. These stresses can result in accumulation of various substances and induce plant-related gene expression and metabolic pathways to strengthen plant resistance. Recently, many genes encoding stress-related proteins have been discovered, and miRNAs have been shown to have important regulatory roles in the expression of these genes (Sanan-Mishra et al., 2009; Baxter et al., 2014; Liu et al., 2015; Zhang and Wang, 2015). Many plant miRNAs can be induced after infection by pathogens, and these miRNAs can participate in plant disease resistance by interacting with their targets (Thiebaut et al., 2015). miR393 was the first miRNA reported to participate in the interaction between plants and pathogens (Navarro et al., 2006). In *Arabidopsis thaliana*, miR824, miR843, miR852, miR166, miR156, and miR159 could respond to *Pseudomonas syringae* (Zhang et al., 2011). Overexpression of miR482b in tomato reduced resistance to *Phytophthora infestans* by inhibiting expression of *NBS-LRR*, while silencing miR482b increased resistance (Jiang et al., 2018). In cucumber, several studies have identified many miRNAs that are related to growth and stress responses (Martínez et al., 2011; Mao et al., 2012; Li et al., 2014; Jin and Wu, 2015; Burkhardt and Day, 2016), but there are currently no reports of miRNAs related to resistance to *C. cassiicola*.

After infection by pathogens, plants will produce secondary metabolites to resist pathogen invasion (Pateraki and Kanellis, 2010; Pusztahelyi et al., 2015). In plants, there are two main types of secondary metabolites that affect disease resistance. One is the inherent substances of plants, such as lignin, callose, and keratin. These substances can reinforce cell walls and prevent pathogens from damaging plant tissues (Zhao and Dixon, 2011). The other group includes alkaloids, terpenes, and phenols that are induced by pathogens. These substances have a direct bactericidal effect (Agrawal and Weber, 2015). Lignin is an important component of the cell wall, with a complex structure and induced properties. Increasing the lignin content can enhance the ability of plant cells to resist penetration and dissolution and inhibit the spread of pathogenic bacteria in plants (Zhao and Dixon, 2011; Li et al., 2015). *Phenylalanine ammonia-lyase* (*PAL*) and *4-coumarate: CoA ligase* (*4CL*) are the two key genes involved in phenylpropanoid synthesis. They can promote the synthesis of lignin to enhance plant disease resistance (Kim and Hwang, 2014; Li et al., 2015).

In our previous study, high-throughput sequencing was performed to investigate the differentially expressed miRNAs in cucumber inoculated with *C. cassiicola*, including two known miRNAs (miR164d and miR396b) and two novel miRNAs (Novel-miR1 and Novel-miR7) (Wang et al., 2018). Based on the analyses of target genes function, we believe that these miRNAs play important roles in the interaction between cucumber and *C. cassiicola*. For further elucidation of cucumber miRNAs, it is necessary to effectively and accurately demonstrate the interaction of miRNAs and target genes. In this study, candidate miRNAs and target genes were transiently expressed in tobacco, and the interaction between miRNAs and target genes was determined by analysis of GUS histochemical staining and fluorescence quantification. Meanwhile, the candidate miRNAs and target genes were transiently expressed in the cucumber cotyledons to investigate the resistance of the transgenic cucumber to *C. cassiicola*. We analyzed the mechanism of cucumber resistance to *C. cassiicola* based on the regulation of miRNAs, which provided a theoretical reference for further stable genetic transformation and breeding research of plants.

Due to the short size of miRNAs and the redundancy of family functions, traditional gene silencing methods are not suitable for miRNA research. Target mimics (TMs) can block the inhibition of target genes by miRNAs, thereby inhibiting the regulatory function of miRNAs (Franco-Zorrilla et al., 2007; Meng et al., 2012). Tang et al. (2012) discovered a new miRNA silencing regulatory mechanism, short tandem target mimic (STTM), which has high silencing efficiency and can be widely used in the functional studies of miRNAs. The STTM is composed of two TMs and a 48 nt linkage sequence. There are three nucleotides forming a bulge between the 10th and 11th nucleotides of the TM on both sides of the STTM, and thus, the binding sites can capture miRNAs without being cleaved by them. Compared to TM, STTM has a better inhibitory effect on miRNAs (Yan et al., 2012). Virus-induced gene silencing (VIGS) can induce plant endogenous gene silencing and alter the plant phenotype to explore gene function (Sha et al., 2014). Tobacco rattle virus (TRV) is an RNA virus that can infect a variety of plants. TRV-based vectors have mild infection symptoms and high gene silencing efficiency (MacFarlane et al., 1999). TRV-based vectors have been widely used in VIGS to inhibit gene expression in a variety of plants (Bachan and Dinesh-Kumar, 2012; Huang et al., 2012; Zhou et al., 2012). However, virus-based miRNA silencing (VBMS) has not been reported in cucumber. In this study, a TRV-based VBMS system that can effectively inhibit the activity of endogenous miRNAs in cucumber for a certain period of time was developed and may provide a strategy for further analysis of the resistance mechanism of cucumber to *C. cassiicola*.

## Materials and Methods

### Plant Materials

*Nicotiana benthamiana* (*N*. *benthamiana*) were planted in a greenhouse at 25°C under 16:8 light/dark cycles for 20 days for agroinfiltration. The cucumber variety used in the experiments was Xintaimici, which was planted in a greenhouse at 28°C under 16:8 light/dark cycles for 10 days for agroinfiltration.

### DNA and RNA Extraction and cDNA Synthesis

The DNA of the cucumber leaves was extracted using a Plant Genomic DNA Kit (Tiangen, Beijing, China). Total RNA was extracted by using a RNAprep Pure Plant Kit (Tiangen, Beijing, China) and synthesized into cDNA using a QuantScript RT Kit (Tiangen, Beijing, China). First-strand cDNA synthesis corresponding to miRNAs was performed using a miRcute miRNA First-Strand cDNA Synthesis Kit (Tiangen, Beijing, China).

### Construction of Transient Expression Vectors of Candidate miRNAs and Their Targets

Based on the precursor sequences of miR164d, miR396b, Novel-miR1, and Novel-miR7 (Supplementary Table S1), primers were designed according to the In-Fusion principles. Similarly, based on the *NAC*, *APE*, *PAL*, and *4CL* open reading frame sequences (Supplementary Table S1), primers were also designed by the In-Fusion principles. The primers were synthesized by GENEWIZ (Suzhou, China) and are listed in Supplementary Table S2. The target genes were annotated via BLAST against the cucumber genome database^1^. The mRNA raw data were deposited in the NCBI Sequence Read Archive (SRA) with the accession number SRP117262; the small RNA raw data were deposited in the NCBI Sequence Read Archive (SRA) with the accession number SRP117230.

pRI-101 AN (TaKaRa, Dalian, China) is a binary vector for plant transformation that can efficiently express exogenous genes. The plant expression vectors pRI-101 AN and pRI-101 AN-GUS were constructed to verify the interaction between miRNAs and targets. For cloning cDNA into the vector, an InFusion HD cloning kit (Clontech, CA, United States) was used. All constructed vectors were confirmed by sequencing before transformation into *Agrobacterium tumefaciens* (*A. tumefaciens*) strain EHA105.

### Co-transformation of Candidate miRNAs and Targets in *N. benthamiana*

To verify the interaction between candidate miRNAs and target genes, we transiently expressed constructed vectors in *N. benthamiana* leaves by *A. tumefaciens* transformation. The agrobacteria carrying constructs were diluted to OD_600_ = 0.5 with suspension buffer (10 mM MES, 10 mM MgCl_2_ and 200 μM acetosyringone). pRI-101 AN-miRNA and pRI-101 AN-GUS-target genes were mixed in equal volumes to test the cleavage function of tae-miR408. Normal tobacco (Control) and tobacco injected with pRI-101 AN-GUS were used as controls. After 48 h of infiltration, GUS staining and fluorescence quantitative analysis were performed as described by Bradford (1976) and Jefferson et al. (1987). All experiments were performed with three biological repeats.

### Transient Expression in Cucumber Cotyledons and Disease Resistance Assays

Transient expression in cucumber cotyledons was performed as described by Shang et al. (2014). The agrobacteria carrying constructs were diluted to OD_600_ = 0.4 with suspension buffer (10 mM MES, 10 mM MgCl_2_ and 200 μM acetosyringone) for cotyledon infiltration. We overexpressed miRNA and target genes to verify disease resistance. Leaves were collected 48 h after agroinfiltration to analyze the expression of miRNAs and target genes by qRT-PCR.

For disease resistance assays, inoculation of *C. cassiicola* was performed by pipetting multiple 10 μL droplet spore suspensions (2 × 10^5^ sporangia/mL) onto cucumber cotyledons. The inoculated samples were kept at 100% relative humidity for 5 days, and disease was assessed by measuring lesion size and quantifying fungal biomass by qRT-PCR quantification of *C. cassiicola Actin*. The primers used were CoActin-F and CoActin-R (Supplementary Table S2). All experiments were performed with three biological repeats. Normal cucumber (Control) and cucumber injected with pRI-101 AN were used as controls. All experiments were performed with three biological repeats.

### Construction of the TRV-Induced Silencing Vector

The sequences of STTM-miR164d, STTM-miR396b, STTM-Novel-miR1, and STTM-Novel-miR7 used in this study were synthesized by GENEWIZ (Suzhou, China). The STTM primers were designed according to the In-Fusion principles and are listed in Supplementary Table S2. The pTRV vector can be efficiently expressed in plants. For cloning STTM into the vector, an InFusion HD cloning kit (Clontech, CA, United States) was used. All constructed vectors were confirmed by sequencing before transformation into *A. tumefaciens* strain EHA105.

### Virus-Based MicroRNA Silencing (VBMS)

Cucumber cotyledons grown for approximately 10 days were used for VBMS, and the suspension method of *Agrobacterium* was the same as described previously. The mixed suspension containing pTRV1 and pTRV2-STTM was injected into the cucumber cotyledons by a sterile syringe. Cotyledons at 7 days post-injection were used for *C. cassiicola* inoculation and disease resistance assays.

### Quantitative Real-Time RT-PCR Assay

qRT-PCR analyses of genes were conducted using a SuperReal PreMix Plus Kit (SYBR Green) (Tiangen, Beijing, China), and the cucumber *Actin* gene was used as the internal control. qRT-PCR analyses of miRNAs were performed using the miRcute miRNA qRT-PCR Detection Kit (SYBR Green) (Tiangen, Beijing, China), and the U6 snRNA was used as a reference to normalize the data. All qRT-PCR assays were performed on a LightCycler 480 system (Roche, CA, United States), and the primers used are listed in Supplementary Table S2. The relative expression was calculated by the 2^-ΔΔCt^ method (Livak and Schmittgen, 2001), and the standard deviation was calculated with three biological repeats.

### Determination of Lignin in Cucumber Cotyledons

The determination of lignin content was performed as described by Morrison (1972) with slight modifications. The lignin content is defined as the absorbance at 280 nm per gram of fresh weight. The 1 g cucumber cotyledon sample was homogenized with 95% ethanol and centrifuged at 5000 rpm for 5 min, and the sediments were washed three times with 95% ethanol. After the samples were washed twice with a mixture of ethanol and n-hexane (1:2, v/v), sediments were collected by centrifugation. The dried samples were dissolved in 2 mL of bromoacetyl and glacial acetic acid (1:3, v/v) solution. After a 30 min water bath at 70°C for 30 min, 0.9 mL 2 mM NaOH was added to stop the reaction. Then, 2 mL glacial acetic acid and 0.1 mL 7.5 M hydroxylamine hydrochloride were added to the sample, and the sample was diluted to 5 mL with glacial acetic acid. After centrifugation at 5000 rpm for 5 min, the supernatants were collected to determine the absorbance at 280 nm.

### Statistical Analysis

All data are the mean (±SD) of three biological replicates. Statistical analysis was carried out by one-way analysis of variance (ANOVA) using the IBM SPSS Statistics 22 software, and the significant differences were determined by Duncan’s multiple range test (*P* < 0.05) and indicated in alphabetical notation.

## Results

### Validation of the Interaction Between Candidate miRNAs and Target Genes

**FIGURE 1:**
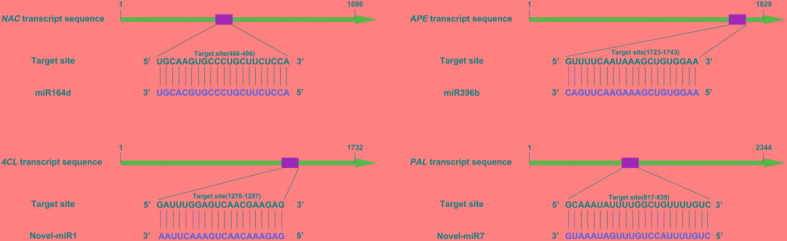
Target sites of candidate miRNAs.

Our previous research found that secondary metabolism plays an important role in cucumber in response to *C. cassiicola* infection (Wang et al., 2018). Based on previous study, two known miRNAs and two novel miRNAs with their targets were selected, as they may be involved in enhanced *C. cassiicola* resistance in cucumber (Table 1). In plants, miRNAs regulate their target gene mainly by recognizing a specific site on the target gene mRNA and binding to it to form a silencing complex, thereby inhibiting translation of the target gene mRNA. Candidate miRNAs and target gene binding sites are shown in Figure 1.

**Table 1:** Candidate miRNAs and their targets.

MicroRNA	Targets ID	Targets description
miR164d	Cucsa.040380	NAC domain containing protein (NAC)
miR396b	Cucsa.098530	Anthranilate phosphoribosyltransferase (APE)
Novel-miR1	Cucsa.261210	4-coumarate: CoA ligase (4CL)
Novel-miR7	Cucsa.124480	Phenylalanine ammonia-lyase (PAL)


**FIGURE 2:**
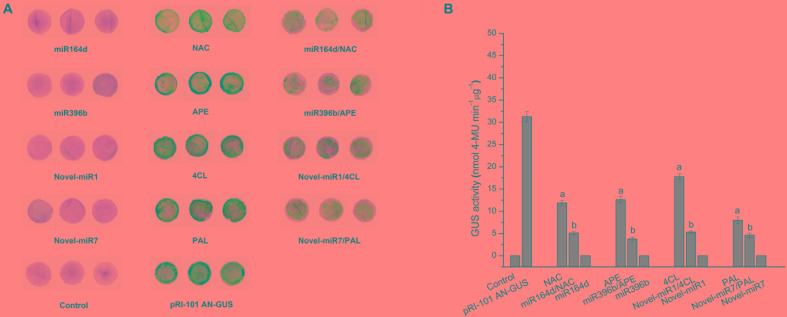
GUS assay of transiently transformed tobacco leaves. **(A)** GUS accumulation by histochemical staining. **(B)** GUS fluorescence quantitative assay. Significance was determined by Duncan’s multiple range test (*P* < 0.05).

To verify the interaction, we co-transformed candidate miRNAs and target genes into tobacco leaves. Using the vector pRI-101 AN-GUS containing the *GUS* gene, we tested the inhibitory effect of miRNAs on target genes with GUS histochemical staining and fluorescence quantification. Normal tobacco (Control) and tobacco inoculated with pRI-101 AN-GUS were used as controls. The phenotypes of GUS histochemical staining are shown in Figure 2A. The GUS phenotypes were not observed in tobacco leaves inoculated with the recombination vector pRI-101 AN-miRNA (miR164d, miR396b, Novel-miR1, and Novel-miR7) and normal tobacco (Control). The GUS phenotypes were observed in tobacco leaves inoculated with pRI-101 AN-GUS, while leaves inoculated with pRI-101 AN-GUS-target gene (*NAC*, *APE*, *4CL*, and *PAL*), in which the target gene was fused upstream of the *GUS* gene, showed similar phenotypes. However, GUS phenotypes were markedly reduced in leaves co-transformed with pRI-101 AN-miRNA (miR164d, miR396b, Novel-miR1, and Novel-miR7) and pRI-101 AN-GUS-target gene (*NAC*, *APE*, *4CL*, and *PAL*).

The GUS protein activity in leaves inoculated with different recombinant vectors was measured by fluorescence quantitative assays (Figure 2B). GUS fluorescence was not detected in normal tobacco and leaves inoculated with pRI-101 AN-miRNA (miR164d, miR396b, Novel-miR1, and Novel-miR7). There were increased GUS fluorescence values in leaves inoculated with pRI-101 AN-GUS and pRI-101 AN-GUS-target genes (*NAC*, *APE*, *4CL*, and *PAL*). The GUS fluorescence values of tobacco leaves co-transformed by pRI-101 AN-miRNA (miR164d, miR396b, Novel-miR1, and Novel-miR7) and pRI-101 AN-GUS-target gene (*NAC*, *APE*, *4CL*, and *PAL*) were determined. The quantitative GUS fluorescence analysis supported the results of GUS histochemical staining. These experiments indicated the existence of a negative regulatory relationship between candidate miRNAs and target genes.

### Transient Expression Levels of Candidate miRNAs and Target Genes

**FIGURE 3:**
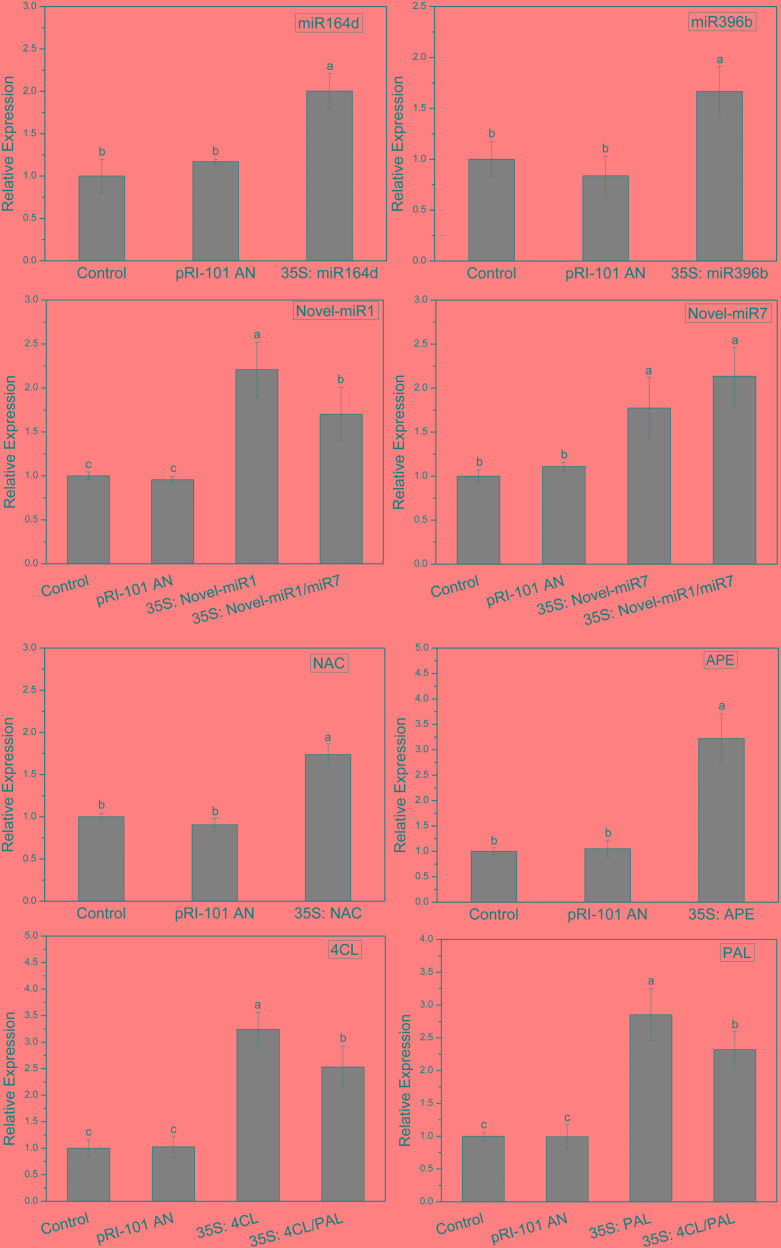
Transient expression levels of miRNAs and target genes in cucumber cotyledons. Significance was determined by Duncan’s multiple range test (*P* < 0.05).

In this experiment, miR164d, miR396b, Novel-miR1, Novel-miR7, Novel-miR1/Novel-miR7 (1:1, v/v), *NAC*, *APE*, *4CL*, *PAL*, and *4CL/PAL* (1:1, v/v) were transiently expressed in cucumber cotyledons via *Agrobacterium* infiltration. Normal tobacco (Control) and tobacco injected with pRI-101 AN were used as controls. Expression levels of candidate miRNAs and target genes in transgenic cucumbers were detected by qRT-PCR. The results are shown in Figure 3. The gene expression levels were similar in the normal tobacco and pRI-101 AN experimental groups. The gene expression level of the overexpression group was significantly higher than that of the controls, which proved that the transient transformation was successful and could be used for further experiments.

### Function of Candidate miRNAs and Target Genes in the Interaction Between Cucumber and *C. cassiicola*

**FIGURE 4:**
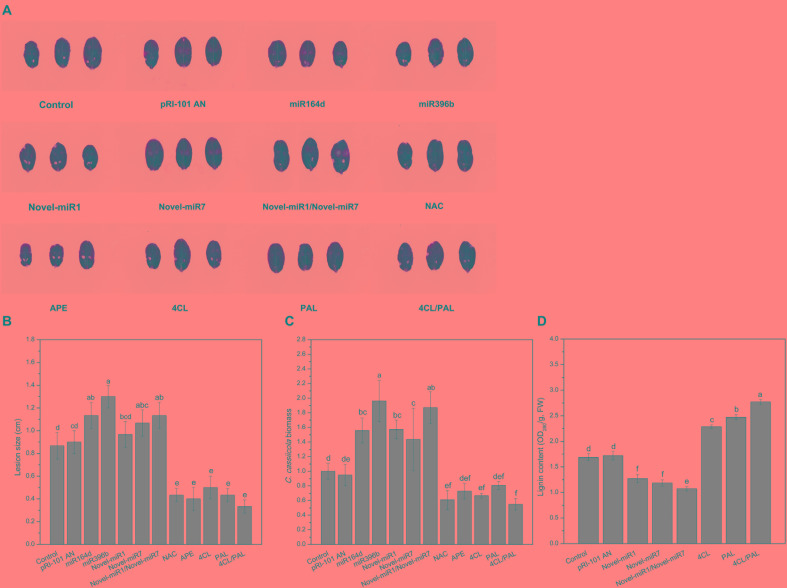
Disease phenotype in cucumber cotyledons transiently expressing miRNAs and target genes after inoculation with *C. cassiicola*. **(A)** Disease symptoms of cucumber cotyledons. **(B)** Disease lesion size of cucumber cotyledons. **(C)** Biomass of *C. cassiicola* of cucumber cotyledons. **(D)** Effect of transient expression of miRNAs and targets on lignin content in cucumber cotyledons. Significance was determined by Duncan’s multiple range test (*P* < 0.05).

We performed analyses of transient expression in cucumber cotyledons to explore the functions of candidate miRNAs and target genes in disease resistance to *C. cassiicola*. Normal cucumber cotyledons (Control) and cucumber cotyledons injected with pRI-101 AN were used as controls. Two days after agroinfiltration for transient expression, the agroinfiltrated cotyledons were collected for disease assays by dropping spore suspensions of *C. cassiicola* onto cucumber cotyledons. The disease phenotype at 5 days after inoculation showed that the *C. cassiicola*-induced lesions in the experimental groups of miR164d, miR396b, Novel-miR7, and Novel-miR1/Novel-miR7 were significantly larger than those in the controls (Figure 4A,B), indicating that transient expression of these miRNAs in cucumber cotyledons reduced the resistance to *C. cassiicola*. However, the *C. cassiicola*-induced lesions on *NAC*-, *APE*-, *4CL*-, and *4CL*/*PAL*-infiltrated leaves were markedly smaller (Figure 4A,B) than those of the controls, showing that transient expression of these genes in cucumber cotyledons improved the resistance to *C. cassiicola*. Expression of the *C. cassiicola Actin* gene was used as a standard of fungal growth. The growth of *C. cassiicola* in the miR164d-, miR396b-, Novel-miR1-, and Novel-miR7-infiltrated leaves was significantly higher than that of the control, while the growth in the *NAC*-, *APE*-, *4CL*-, and *4CL*/*PAL*-infiltrated leaves was markedly lower than that of the control (Figure 4C). In addition, the disease lesions of the Novel-miR1 group did not increase significantly, but the growth of *C. cassiicola* increased significantly (Figure 4A–C). Transient expression assays showed that overexpression of candidate miRNAs could reduce the resistance to *C. cassiicola*, and overexpression of target genes corresponding to candidate miRNAs could improve the resistance to *C. cassiicola*. These results were consistent with those of our previous analysis.

The previous analysis shows that Novel-miR1 and Novel-miR7 can inhibit *4CL* and *PAL*, respectively. The *4CL* and *PAL* genes are two key genes in the lignin synthesis pathway, and the lignin content is positively correlated with disease resistance. In this experiment, Novel-miR1, Novel-miR7, Novel-miR1/Novel-miR7 (1:1, v/v), *4CL*, *PAL*, and *4CL/PAL* (1:1, v/v) were transiently expressed in cucumber cotyledons through agroinfiltration. Normal tobacco (Control) and tobacco injected with pRI-101 AN were used as controls. Two days after agroinfiltration for transient expression, the agroinfiltrated cotyledons were prepared for determination of lignin content (Figure 4D). The lignin content was determined to further analyze the effect of overexpression of candidate miRNAs and target genes on lignin accumulation in cucumber cotyledons. As shown in Figure 4D, the lignin content was significantly increased in *4CL*-, *PAL*-, and *4CL*/*PAL*-infiltrated samples compared to that of the control, and the lignin content in Novel-miR1-, Novel-miR7-, and Novel-miR1/Novel-miR7-infiltrated leaves was obviously decreased compared to that of the control. The lignin content of *4CL*/*PAL*-infiltrated leaves was the highest, while the lignin content of Novel-miR1/Novel-miR7-infiltrated leaves was the lowest. The results showed that overexpression of *4CL* and *PAL* could increase the lignin content in cucumber leaves, and overexpression of Novel-miR1 and Novel-miR7 could reduce lignin in cucumber leaves.

### TRV-Induced VBMS

**FIGURE 5:**
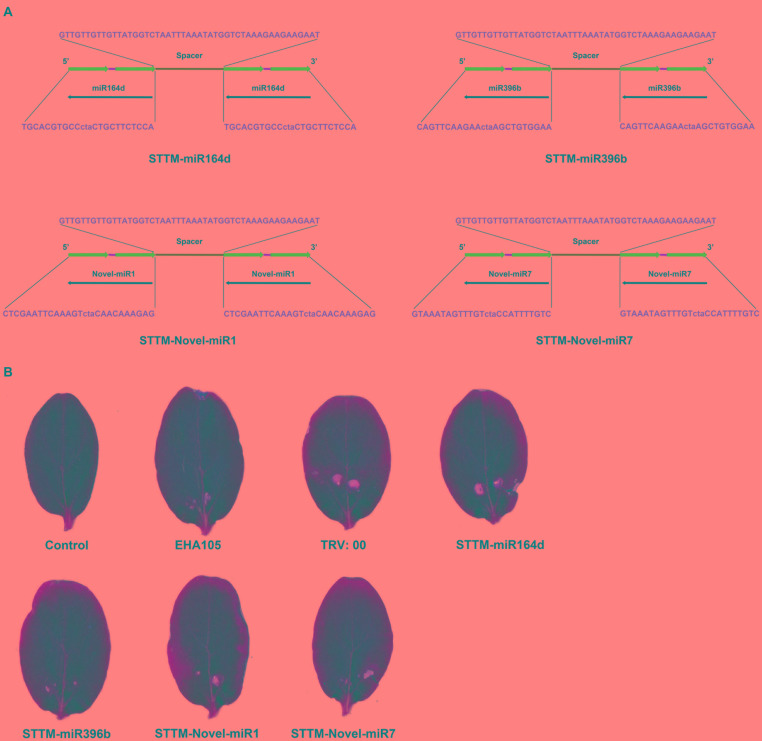
TRV induced STTM-miRNA silencing. **(A)** Diagram of STTM. **(B)** Symptoms in cucumber cotyledons following TRV-induced genes silencing.

In this study, STTM-miR164d, STTM-miR396b, STTM-Novel-miR1, and STTM-Novel-miR7 recombinant vectors were constructed based on TRV. The STTM structures are shown in Figure 5A. The sequences of STTM-miR164d, STTM-miR396b, STTM-Novel-miR1, and STTM-Novel-miR7 used in this study are listed in Supplementary Table S1. The sides of the structure are TMs, and the restriction sites at both ends are *Eco*RI and *Sac*I. STTM and pTRV2 were used to construct recombinant vectors by In-Fusion technology. Recombinant viruses TRV: 00 (pTRV1+pTRV2), TRV: STTM-miR164d, TRV: STTM-miR396b, TRV: STTM-Novel-miR1, and TRV: STTM-Novel-miR7 were infiltrated into cucumber cotyledons. After inoculation for 7 days, chlorosis and a few virus spots appeared in the cotyledons of cucumber inoculated with TRV recombinant virus, but no obvious phenotype was observed on the cotyledons of Control and *A. tumefaciens* EHA105 (Figure 5B), indicating that TRV had successfully replicated and proliferated in the cotyledons of cucumber.

**FIGURE 6:**
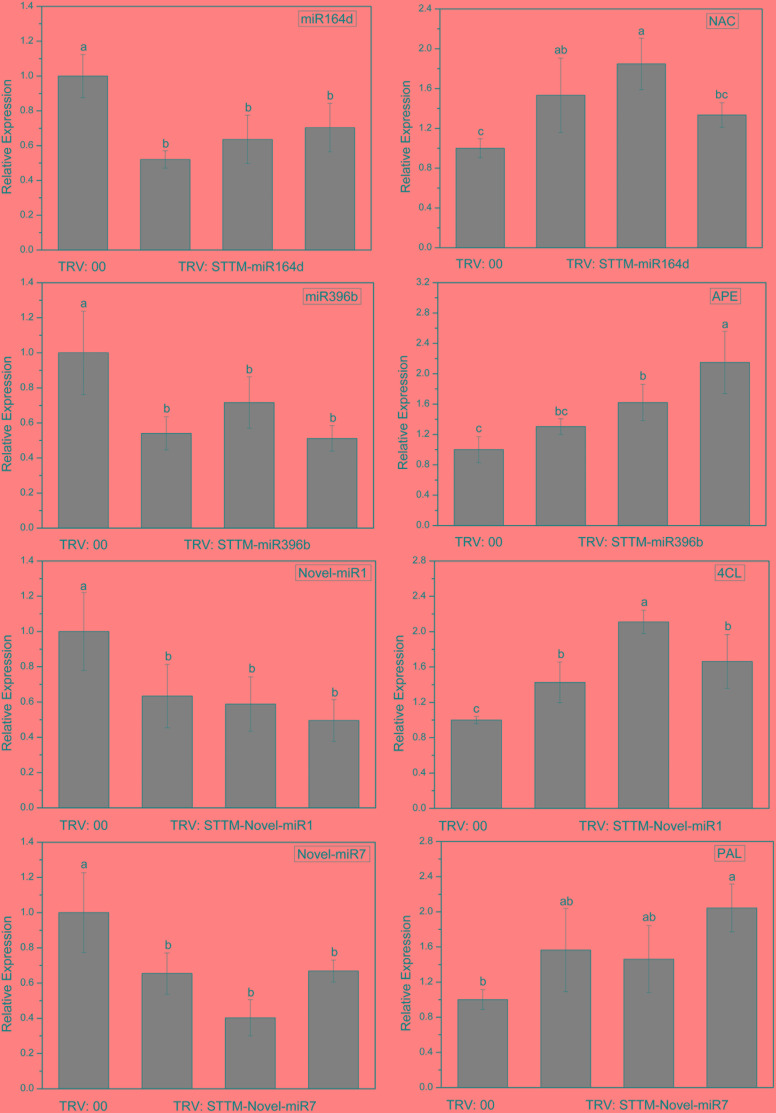
Expression levels of miRNAs and targets in cucumber cotyledons. Significance was determined by Duncan’s multiple range test (*P* < 0.05).

To explore the silencing efficacy of candidate miRNAs, we detected the expression levels of candidate miRNAs and corresponding target genes in cucumber cotyledons injected with TRV: STTM by qRT-PCR. As shown in Figure 6, the expression levels of candidate miRNAs in the cotyledons infiltrated with TRV: STTM-miR164d, TRV: STTM-miR396b, TRV: STTM-Novel-miR1, and TRV: STTM-Novel-miR7 were significantly decreased, and the expression levels of target genes corresponding to these miRNAs were upregulated. These results indicated that candidate miRNAs in cucumber cotyledons were successfully inhibited.

### Response of Cucumber Cotyledons to *C. cassiicola* After Candidate miRNAs Silencing

**FIGURE 7:**
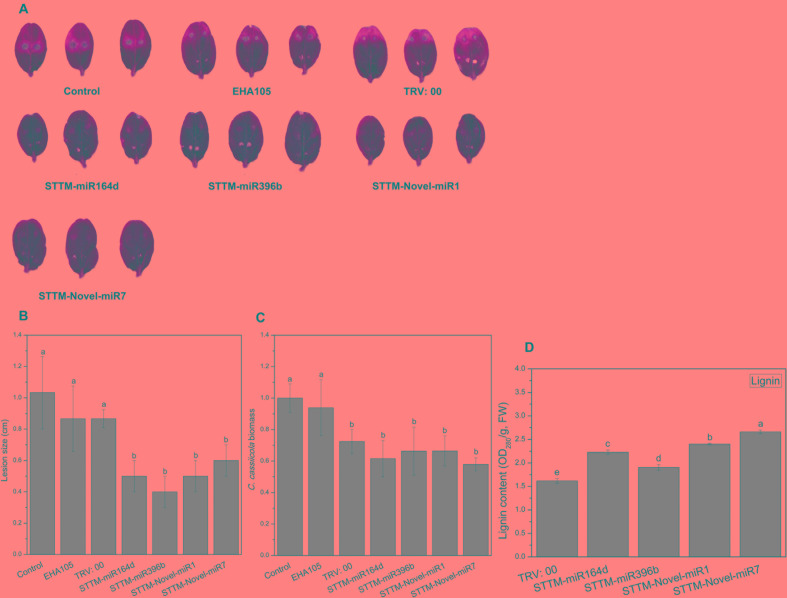
Disease phenotype in STTM transgenic cucumber cotyledons after inoculation with *C. cassiicola*. **(A)** Disease symptoms of cucumber cotyledons. **(B)** Disease lesion size of cucumber cotyledons. **(C)** Biomass of *C. cassiicola* of cucumber cotyledons. **(D)** Effect of candidate miRNAs silencing on lignin content in cucumber cotyledons. Significance was determined by Duncan’s multiple range test (*P* < 0.05).

To identify the role of STTM-miRNA in the interaction between cucumber and *C. cassiicola*, we inoculated the TRV: 00 (pTRV1+pTRV2)-, TRV: STTM-miR164d-, TRV: STTM-miR396b-, TRV: STTM-Novel-miR1-, and TRV: STTM-Novel-miR7-infiltrated cucumber cotyledons with *C. cassiicola* to observe phenotypic changes. After 5 days of inoculation of *C. cassiicola*, there was no significant difference between the disease lesions of TRV: 00 (pTRV1+pTRV2)-infiltrated cotyledons and control cotyledons. However, the *C. cassiicola*-induced lesions of miR164d-, miR396b-, Novel-miR1-, and Novel-miR7-silenced cucumber cotyledons were markedly smaller (Figure 7A,B) than those of the control. The biomass of *C. cassiicola* in the infected cotyledons of cucumber was detected by qRT-PCR (Figure 7C), and the expression of the *C. cassiicola Actin* gene in candidate miRNA-silenced cucumber cotyledons decreased significantly. Thus, silencing of miR164d, miR396b, Novel-miR1 and Novel-miR7 increased cucumber resistance to *C. cassiicola*. The lignin content was determined to further analyze the effect of silencing candidate miRNAs on lignin accumulation in cucumber cotyledons (Figure 7D). The lignin content was significantly upregulated in all silenced plants, especially in Novel-miR1- and Novel-miR7-silenced plants. Because *4CL* (inhibited by Novel-miR1) and *PAL* (inhibited by Novel-miR7) are upstream and downstream genes in the phenylpropane pathway, which is related to lignin synthesis, this finding is also consistent with our previous experimental results.

## Discussion

As miRNAs have no coding function, they can only act by inhibiting or degrading the corresponding targets. Genes related to abiotic and biotic stresses have been proven to be targets of miRNAs and can be used to determine the function of miRNAs (Jagadeeswaran et al., 2009; Katiyar-Agarwal and Jin, 2010). In plants, the interaction between miRNAs and target genes was verified by 5′ RNA ligase-mediated rapid amplification of cDNA ends (5′ RLM-RACE) (Llave et al., 2002). The RACE method is cumbersome and does not visualize the interaction between the miRNA and target gene. *Agrobacterium*-mediated transient expression of tobacco is highly efficient and has a long expression time, which is suitable for the study of gene interactions in plants (Prabu and Prasad, 2012; Yin et al., 2017). This method is mainly used to verify the interaction between miRNAs and target genes by detecting the expression of the *GUS* gene in tobacco after transient expression of miRNAs and target genes (Feng et al., 2013; Han et al., 2016).

NAC is a plant-specific transcription factor that is involved in plant development and stress responses in plants. Previous studies have shown that many members of the *NAC* gene family exhibit differential expression characteristics after pathogen infection in plants, which indicates that *NAC* genes have specific biological functions in response to disease resistance in plants (Jensen and Skriver, 2014; Qin et al., 2014). In a recent study, researchers analyzed cucumber *NAC* genes related to the response to abiotic stresses (Zhang et al., 2017). However, there are few studies on the role of *NAC* genes in the interaction between cucumber and pathogens. BLAST analysis of existing data showed that the *NAC* gene we identified was *csNAC30*. Some studies have shown that *NAC* affects the synthesis of secondary metabolites, but the detailed mechanism is not clear (Zhao et al., 2010; Xu et al., 2015).

Tryptophan not only promotes endogenous jasmonic acid (JA) biosynthesis and triggers plant signal transduction pathways but also participates in the terpenoid indole alkaloid (TIA) pathway, which in turn regulates the plant response to stress (Sun et al., 2016). Anthranilate phosphoribosyltransferase (APE) is an important enzyme in the tryptophan synthesis pathway, which is indirectly involved in the synthesis of plant secondary metabolites (Dharmawardhana et al., 2013).

*Phenylalanine ammonia-lyase* (*PAL*) and *4-coumarate: CoA ligase* (*4CL*) are two key genes in the phenylpropane synthesis pathway and are closely related to plant resistance to external stresses. First, PAL catalyzes phenylalanine to cinnamic acid; then, cinnamic acid participates in the synthesis of ubiquinone and is catalyzed by 4CL to form cinnamoyl-CoA; finally, cinnamoyl-CoA further synthesizes lignin and flavonoids (Kim and Hwang, 2014; Li et al., 2015).

After analyzing the function of the target genes, we selected miR164d, miR396b, Novel-miR1, and Novel-miR7 and their target genes *NAC*, *APE*, *4CL*, and *PAL* for interaction verification. Histochemical staining and quantitative detection of *GUS* genes showed that these target genes could be suppressed by the corresponding miRNA, but the inhibition efficiency was not 100%. There are two possible causes for this result. First, there is a balance between the transcription of target genes and nucleotide cleavage, and this balance is affected by the expression level of miRNAs (Nikovics et al., 2006). Second, it may be that only a portion of the target mRNA was cleaved and degraded by the miRNA, and the remaining target mRNAs were detached from the cleavage system and normally transcribed (Adam et al., 2010).

**FIGURE 8:**
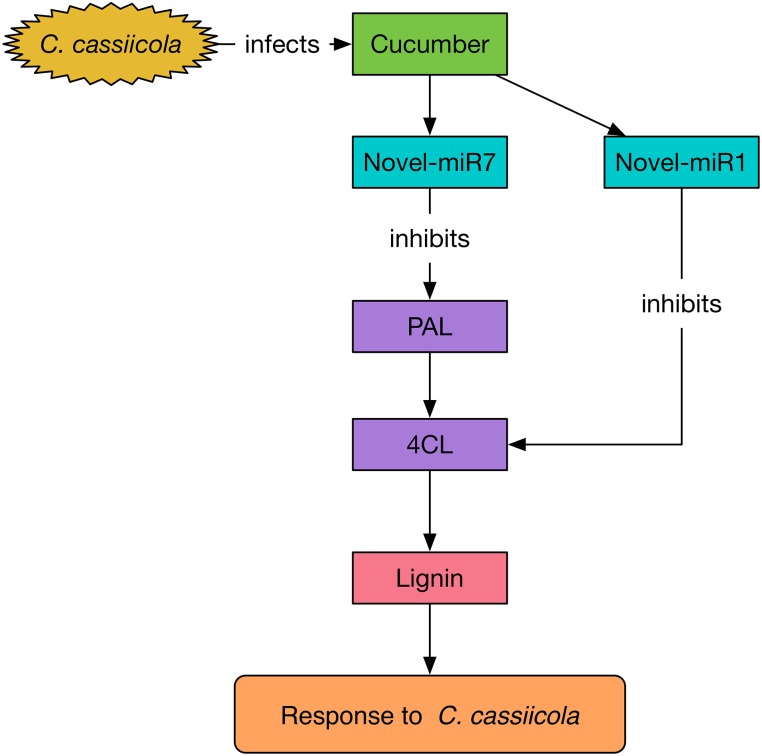
Mechanism of Novel-miR1 and Novel-miR7 regulation network involved in the response to *C. cassiicola*.

To explore the functions of miR164d, miR396b, Novel-miR1, and Novel-miR7 and their target genes *NAC*, *APE*, *4CL*, and *PAL* in cucumber response to *C. cassiicola*, we transiently expressed candidate miRNAs and target genes in cucumber cotyledons. After vaccination with *C. cassiicola*, phenotypic changes were observed. Because *4CL* and *PAL* are upstream and downstream genes in the phenylpropanoid metabolic pathway, we coexpressed *4CL* and *PAL* to establish the experimental group. Similarly, Novel-miR1 and Novel-miR7 were also coexpressed. Figure 4 shows that the disease resistance of the target gene overexpression groups was significantly higher than that of the control groups, indicating that these genes play an important role in cucumber resistance to *C. cassiicola*. The disease resistance of the candidate miRNAs overexpression group was lower than that of the control group, probably because these miRNAs inhibited the expression of their target genes. These results were also confirmed by measurement of the biomass of *C. cassiicola* in cucumber cotyledons after infection. The data showed that the coexpressed *4CL* and *PAL* groups had the highest disease resistance, and the disease resistance of the corresponding Novel-miR1 and Novel-miR7 coexpression groups was the lowest. Because the phenylpropanoid metabolic pathway affects the synthesis of lignin, and *4CL* and *PAL* are important genes in this pathway, we performed lignin content determination after transient expression of candidate miRNAs and genes in cucumber cotyledons for 2 days. Figure 4D shows that the lignin content of *4CL*/*PAL*-infiltrated leaves was the highest; the lignin content of Novel-miR1-, Novel-miR7-, and Novel-miR1/Novel-miR7-infiltrated leaves was lower than that of the normal tobacco, and the lignin content reduction was more severe in the Novel-miR1/Novel-miR7-infiltrated leaves. STTM is an effective tool for studying miRNA function in plants and animals. Tang et al. (2012) developed STTM technology based TM. Overexpression of STTM has been used to identify the functions of miRNAs in a variety of plants, such as *Arabidopsis* and wheat (Jia et al., 2015; Jiao et al., 2015), but it has not been reported in cucumber. To further explore the function of candidate miRNAs, we developed a TRV-induced VBMS to silence cucumber endogenous miRNAs. The results showed that silencing of miR164d, miR396b, Novel-miR1 and Novel-miR7 increased the resistance of cucumber cotyledons to *C. cassiicola*, indicating that these miRNAs played a negative regulatory role in cucumber resistance to *C. cassiicola*. Notably, lignin content is highest in Novel-miR1- and Novel-miR7-silenced cucumber cotyledons. These findings also indicate that the interaction between Novel-miR1 and Novel-miR7 and their target genes affects the synthesis of lignin, which in turn affects the resistance of cucumber to *C. cassiicola* (Figure 8).

## Conclusion

In our study, the expression vectors were constructed by In-Fusion technology, and the negative relationships of miR164d, miR396b, Novel-miR1, and Novel-miR7 and their targets were verified by the *Agrobacterium*-mediated tobacco transient expression system. Meanwhile, we found that overexpression of *NAC*, *APE*, *4CL*, and *PAL* could improve the resistance to *C. cassiicola*, and overexpression of miR164d, miR396b, Novel-miR1, and Novel-miR7 could reduce the disease resistance in cucumber. We confirmed that silencing candidate miRNAs could improve the disease resistance of cucumber, and the lignin content in Novel-miR1- and Novel-miR7-silenced cucumber cotyledons was significantly increased. These candidate miRNAs and targets are closely related to cucumber lignin synthesis, and the data combined with previous analyses demonstrate the important role of secondary metabolism, especially the lignin metabolism pathway, in the process of cucumber resistance to *C. cassiicola*.

## Data Availability

The datasets generated for this study can be found in NCBI Sequence Read Archive (SRA), SRP 117262 and SRP117230.

## Author Contributions

XW and HF conceived and designed the research. XW performed the experiments and wrote the manuscript. All authors analyzed the data and read and approved the final manuscript. HF revised the manuscript.

## Conflict of Interest Statement

The authors declare that the research was conducted in the absence of any commercial or financial relationships that could be construed as a potential conflict of interest.

## Footnotes

1 https://phytozome.jgi.doe.gov/pz/portal.html
